# Primary Ewing’s sarcoma of sphenoid sinus: A case report and literature review

**DOI:** 10.3389/fonc.2022.894833

**Published:** 2022-08-15

**Authors:** Kunpeng Wu, Xiaoyan Zhu, Yan Li, Daxiong Wen, Huiyu Wu, Yanzhen Lai, Yun Li, Jian Wu, Zhuoxing Liu

**Affiliations:** ^1^ Department of Oncology, Heyuan Hospital of Guangdong Provincial People’s Hospital, Heyuan People’s Hospital, Heyuan, China; ^2^ Department of Otorhinolaryngology, Heyuan Hospital of Guangdong Provincial People’s Hospital, Heyuan People’s Hospital, Heyuan, China; ^3^ Department of Radiology, Heyuan Hospital of Guangdong Provincial People’s Hospital, Heyuan People’s Hospital, Heyuan, China; ^4^ Department of Pathology, Heyuan Hospital of Guangdong Provincial People’s Hospital, Heyuan People’s Hospital, Heyuan, China

**Keywords:** Ewing’s sarcoma, sphenoid sinus, sphenoid bone, case report, literature review

## Abstract

**Background:**

Primary Ewing’s sarcoma of sphenoid sinus, observed in children and adolescents, is an extremely rare malignancy. Such rarity makes the imaging features and treatment strategies for Ewing’s sarcoma of sphenoid sinus unclear. This study aimed to offer guidance for treating this very disease by describing a patient with a rare primary Ewing’s sarcoma of sphenoid sinus and reviewing the available data in the literature.

**Case description:**

A case of Ewing’s sarcoma in sphenoid sinus treated with multidisciplinary treatment approaches, including tumor resection, radiotherapy, chemotherapy, and antiangiogenic therapy, was presented in this study. Moreover, literature for Ewing’s sarcoma in the head was systematically searched, and two cases in the sphenoid sinus and five cases in the sphenoid bone were identified. Furthermore, the clinical features, imaging findings, pathological characteristics, treatment, and prognosis were summarized.

**Conclusion:**

Tumor resection combined with radiotherapy and chemotherapy may provide favorable results for patients with Ewing’s sarcoma of sphenoid sinus and bone. However, more reports are still necessary to further clarify optimal management.

## Introduction

**Figure 1:**
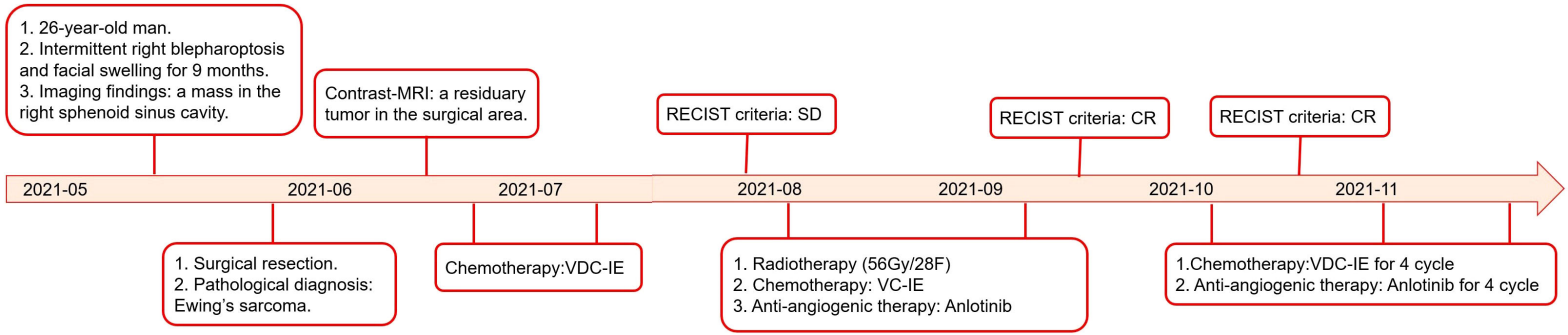
Treatment course of the case in a timeline (MRI: magnetic resonance imaging; CR: complete response; PR: partial response; V: vincristine; D: doxorubicin; C cyclophosphamide; I: ifosfamide; and E: etoposide).

Ewing’s sarcoma is an aggressive bone tumor, and ranks second only to osteosarcoma as the most common malignancy in children and young adults (1). It predominately occurs in the shafts of the long bones, the pelvic bones, ribs, and spine, comprising 70% of all Ewing’s sarcoma cases (2). Primary Ewing’s sarcoma can be rarely detected in the head and neck region, especially in sphenoid sinus (3). Few literatures on the case of primary Ewing’s sarcoma in sphenoid sinus were ever reported. Thus, a case of a 26-year-old male patient with primary Ewing’s sarcoma of sphenoid sinus was presented (**Figure 1**).

## Case report

**Figure 2:**
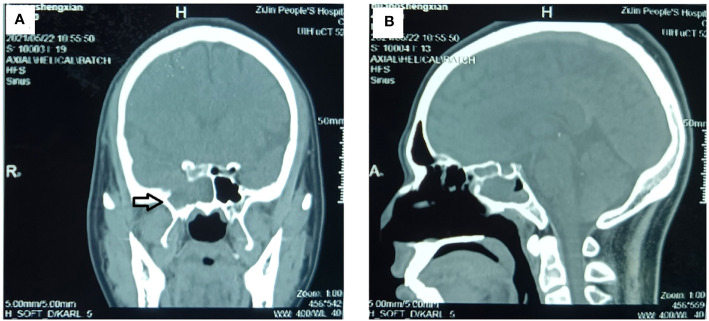
Preoperative CT examination of the paranasal sinus. The CT examination showed an inhomogeneous mass in the right sphenoid sinus cavity, and steolytic bone destruction and defect in the parietal wall of sphenoid sinus. **(A)** Sagittal image and **(B)** coronal image. (The mass is marked with a black arrow.).

**Figure 3:**
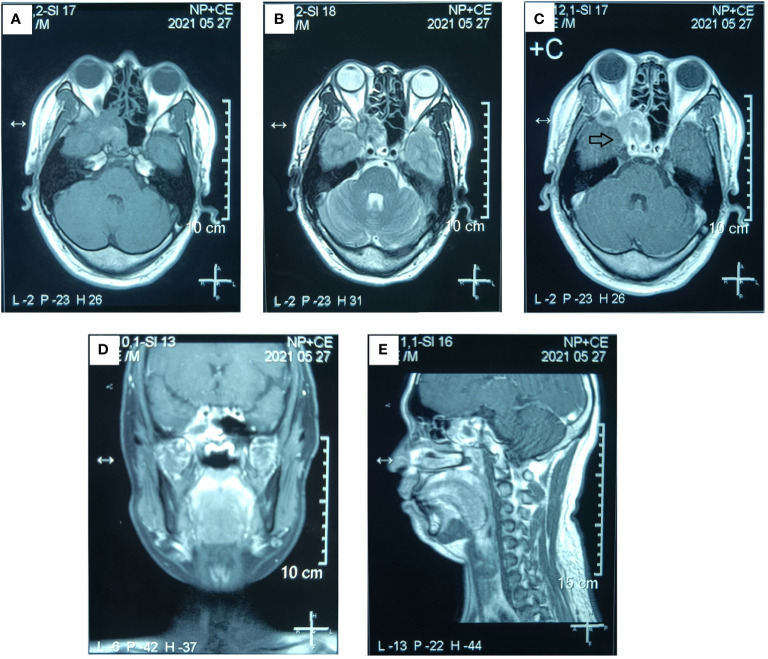
Preoperative MR examination of the paranasal sinus. The MR examination detected a solid mass in the right sphenoid sinus that had extended into the right orbital apex and posterior ethmoid sinus. **(A)** Axial T1-weighted image. **(B)** Axial T2-weighted image. **(C)** Axial T1-weighted enhanced image. **(D)** Sagittal T1-weighted enhanced image. **(E)** Coronal T1-weighted enhanced image. (The mass is marked with a black arrow.).

**Figure 4:**
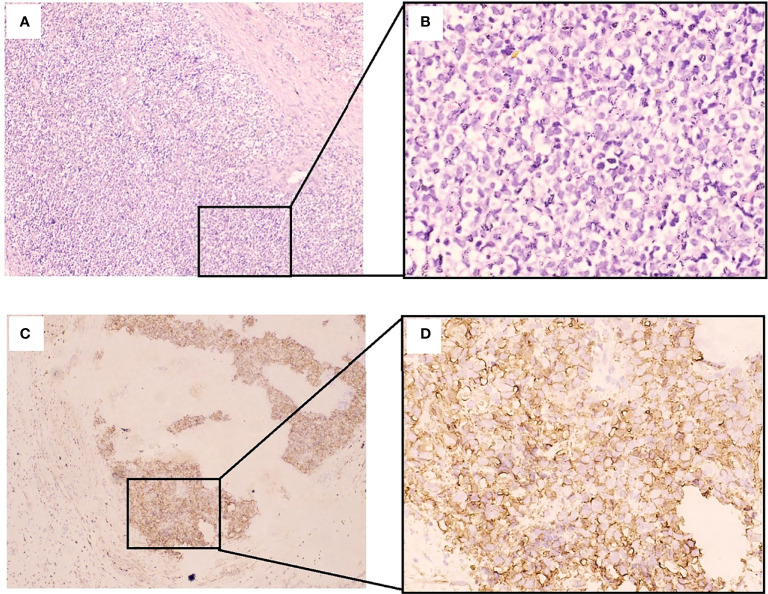
Histological and immunohistochemical features of the tumor of sphenoid sinus. **(A, B)** Hematoxylin and eosin (H & E) staining of a tumor specimen showing small round atypical cells with densely stained chromatin and high nuclear cytoplasmic ratios. **(B, C)** Immunohistochemical staining of a tumor specimen showing diffuse positive staining for CD99 on the tumor cell membrane. **(A, C)**: Original magnification × 100; **B, D**: Original magnification × 400.).

**Figure 5:**
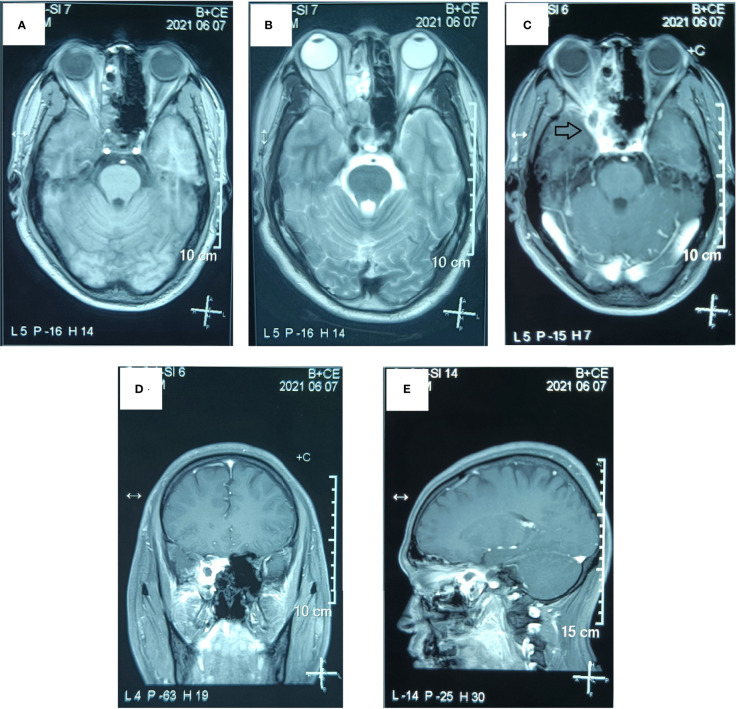
Postoperative MR examination of the paranasal sinus. The MR examination detected a residuary tumor in the surgical area and the lateral side and the tip of the right orbit. **(A)** Axial T1-weighted image. **(B)** Axial T2-weighted image. **(C)** Axial T1-weighted enhanced image. **(D)** Sagittal T1-weighted enhanced image. **(E)** Coronal T1-weighted enhanced image. (The mass is marked with a black arrow.).

In May 2021, a 26−year−old man underwent an otorhinolaryngological clinical examination due to a history of intermittent right blepharoptosis and facial swelling; he had had these symptoms for 9 months. He was detected to have a mildly tender firm swelling in the right temporal region, and the eye movement, visualization, mouth opening, and airway were within normal limits. The patient had a history of occasional smoking, but no trauma, infection, or previous medical history, as well as an unremarkable family history. The blood routine examination, usual biochemical tests, and tumor biomarkers were also within normal limits. The computed tomography (CT) scan showed an inhomogeneous mass in the right sphenoid sinus cavity, and steolytic bone destruction and defect in the parietal wall of sphenoid sinus (**Figure 2**). Contrast magnetic resonance imaging (MRI) was then used to determine the extent of the tumor, and displayed a solid mass in the right sphenoid sinus that had extended into the right orbital apex and posterior ethmoid sinus (**Figure 3**). Contrast material-enhanced CT of the whole body was performed to exclude tumor distant metastasis. Based on preoperative imaging study findings, the otolaryngologist initially considered it a nonmalignant mass. Subsequently, the patient underwent otorhinolaryngological surgical removal of the lesion, when the intraoperative frozen specimen suggested a small round cell tumor, and the diagnosis of malignancy could not be clearly defined. The tumor was partially resected, but given that an attempt at radical resection was associated with high risks of intraoperative massive bleeding and visual impairment, the part of the tumor that adheres severely to the optic nerve sheath and the cavernous sinus was not resected. Postoperative histopathological examination showed that the tumor cells were small oval or round with scanty cytoplasm, and round or oval vesicular nuclei (**Figures 4A, B**). Immunohistochemistry revealed membranous positivity for CD99 (**Figures 4C, D**), diffuse nuclear positivity for NKX2.2, diffuse cytoplasmic positivity for vimentin and sporadic positivity for Syn and EMA, strongly suggesting a diagnosis of Ewing’s sarcoma. In this case, the tumor cells were immunohistochemically negative staining for CK, LCA, CgA, CD56, P40, CK7, CK5/6, IMP3, S100, GFAP, HMB45, and SOX10, respectively. The pathological diagnosis was confirmed by two pathologists. After surgery, his intermittent right blepharoptosis and facial swelling disappeared. The contrast MRI was conducted to evaluate the tumor status, and a residuary tumor was detected in the surgical area (**Figure 5**). The multidisciplinary consultation meeting was conducted for making postoperative courses.

**Figure 6:**
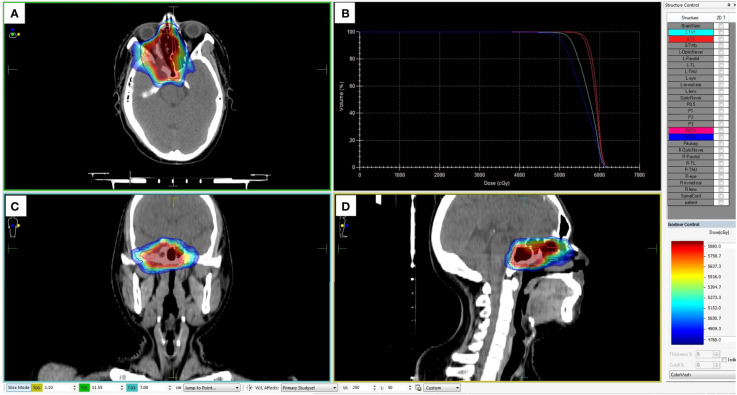
Dose planning of radiotherapy. Planned target volume (GTV: red, PGTV: pink, CTV: light blue, and PTV: blue) covered by the 95% isodose can be seen in axial **(A)**, coronal **(C)**, and sagittal **(D)** planes on pretreatment planning computed tomography. **(B)** The dose-volume histogram showed that 95% of the PGTV and PTV volumes irradiated with more than 5,040 cGy and 5,600 cGy.

**Figure 7:**
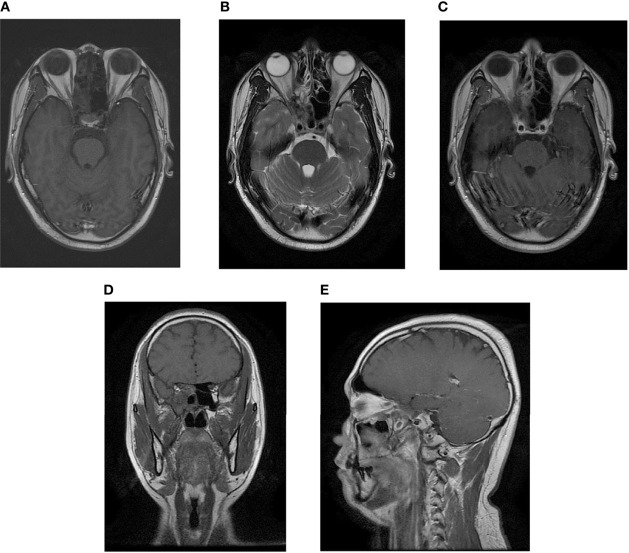
Postradiotherapy MR examination of the paranasal sinus. The MR examination detected a complete remission of the tumor. **(A)** Axial T1-weighted image. **(B)** Axial T2-weighted image. **(C)** Axial T1-weighted enhanced image. **(D)** Sagittal T1-weighted enhanced image. **(E)** Coronal T1-weighted enhanced image.

To reduce the radioactive exposure of the right eye, the patient was arranged to undergo chemotherapy with alternating cycles of doxorubicin (75 mg/m^2^ on day 1), vincristine (2 mg on day 1), cyclophosphamide (1,200 mg/m^2^ on day 1), and ifosfamide (1,800 mg/m^2^ on days 1–5) plus etoposide (100 mg/m^2^ on days 1–5) every 3 weeks before proceeding with the radiotherapy. Unfortunately, the response evaluation criteria in solid tumors (RECIST) criteria confirmed the residuary tumor as a stable disease after two cycles of postoperative chemotherapy. Then, intensity-modulated radiotherapy [gross target volume (GTV) 5,600 centigray (cGy)/28 fractions, clinical target volume (CTV) 5,040 cGy/28 fractions, with details summarized in **Figure 6**] and anti-angiogenic therapy (anlotinib, 10 mg on days 1–14, every 3 weeks) were conducted on the basis of chemotherapy. The patient experienced certain treatment–related toxicities, including anorexia, nausea, vomit, fatigue, diarrhea, leucopenia, neutropenia, alanine aminotransferase concentration increase, and aspartate aminotransferase concentration increase, within grade 3. After 4 weeks of radiotherapy, there was a complete remission of the tumor by the RECIST criteria (**Figure 7**). Twelve months after the diagnosis, the patient remained recurrence−free and regained his high-quality life.

## Discussion

Ewing’s sarcoma was first described as “diffuse endothelioma of bone” by James Ewing in 1921 (4). Generally, the majority of Ewing’s sarcoma is observed in the diaphysis or metadiaphyseal region of long bones. However, primary Ewing’s sarcoma is extremely rare in the head and neck region, accounting for merely 1% to 9% (5–8). In addition, the most common sites for Ewing’s sarcoma of the head and neck are the mandible and skull base, followed by the orbital roof, maxilla, and nasal cavities (5–8). Meanwhile, the most common symptoms are presented as painful mass, swelling, headaches, visual problems, and congestion/obstruction (5–8). Based on retrospective analysis, age, clinical stage, tumor site, and the response to systemic chemotherapy are important prognostic factors for disease-free survival and overall survival in patients with Ewing’s sarcoma of the head and neck (3, 6). Like the treatment for skeletal Ewing’s sarcoma, induction chemotherapy and local control (surgical resection ± radiotherapy) are the frequently adopted treatment approaches for Ewing’s sarcoma in the head and neck, followed by adjuvant chemotherapy (9, 10).

In the present study, a case of primary Ewing’s sarcoma in sphenoid sinus was investigated. Limited by small patient series, there was no retrospective study about Ewing’s sarcoma of sphenoid sinus. The Springer, Elsevier, Wiley, MEDLINE, and EMBASE databases were systematically searched to March 2022. Given that the case of primary Ewing’s sarcoma in sphenoid sinus was rarely reported, a literature review was hereby presented. Only two cases of primary Ewing’s sarcoma in sphenoid sinus (11, 12) and five cases in the sphenoid bone (13–18) were found in the published articles. As shown in **Table 1**, the onset age and sex ratio of these cases resemble the status of overall Ewing’s sarcoma. All cases with primary Ewing’s sarcoma in sphenoid sinus and bone were diagnosed with non-metastatic disease, which might be attributed to early clinical symptoms and limited growth space. Meanwhile, diminution of vision, proptosis, headache, and swelling were found to be the most common symptoms (11–14, 16–18). In addition, a diffuse and strong membranous staining pattern for CD99 was universally considered as a highly sensitive and useful immunohistochemical biomarker for Ewing’s sarcoma (19, 20). In system treatment, surgical excision of the tumor combined with radiotherapy and chemotherapy was considered an appropriate treatment for Ewing’s sarcoma of sphenoid sinus and bone (9, 10), while in local treatment, surgical excision and radiotherapy are the main strategies (21). The limitation of the anatomy of sphenoid bone and sinus makes it difficult for tumor excision to achieve enough safe surgical margins (9, 10). Thus, radiotherapy is considered as an effective postoperative treatment for improving local control (22, 23), which can also be adopted to replace surgical excision for patients unsuitable for surgery (22, 23). Vincristine, isofosfamide/cyclophosphamide, doxorubicin, etoposide, and actinomycin-D are the most frequently used drugs in cytotoxic chemotherapy (24). Based on guidelines of Ewing’s sarcoma available in the 2021 National Comprehensive Cancer Network (NCCN), ifosfamide and etoposide alternating with the standard regimen of vincristine, doxorubicin, and cyclophosphamide (VDC-IE) has become a standard for Ewing’s sarcoma patients, and multiagent chemotherapy should be performed at least 9 weeks prior to local therapy. As shown in **Table 1**, given the lack of preoperative biopsy, preoperative chemotherapy was not conducted in these cases. Interestingly, poor response to chemotherapy was observed in primary Ewing’s sarcoma of sphenoid sinus. Negru et al. showed that the patient with Ewing’s sarcoma of sphenoid sinus was identified as local disease progression by MRI after three cycles of first−line chemotherapy (VID:; vincristine, isofosfamide, and doxorubicin) (11). Furthermore, Turki et al. suggested that the tumor did not respond to chemotherapy (VIDE; vincristine, isofosfamide, doxorubicin, and etoposide) and the patient died after three cycles of chemotherapy (12). In the hereby proposed case, the patient was confirmed to have stable disease after two cycles of postoperative chemotherapy (VDC-IE: vincristine, doxorubicin, and cyclophosphamide, alternating with ifosfamide and etoposide), and radiotherapy was therefore performed, followed by anti-angiogenic therapy (anlotinib, a novel oral receptor tyrosine kinase inhibitor targeting vascular endothelial growth factor receptors 2 and 3, fibroblast growth factors 1–4, platelet-derived growth factor receptor α and β, c-Kit, and Ret, applicable to the treatment of soft-tissue sarcoma in China (25, 26)). The poor chemosensitivity for Ewing’s sarcoma of sphenoid sinus was preliminarily attributed to lack of rich tumor blood supply in comparison to Ewing’s sarcoma of long bones. Indeed, the prognosis for patients with primary Ewing’s sarcoma in sphenoid sinus and bone is still unclear, and patients with Ewing’s sarcoma of the head and neck region tend to have favorable prognosis compared with those with Ewing’s sarcoma of the proximal extremity or axial skeleton (27). It can be observed from **Table 1** that patients capable of completing the treatment plan might achieve adequate long-term prognosis and cure.

**Table 1:** Primary Ewing’s sarcoma of sphenoid sinus and bone in the literature.

Authors	Years	Country	Sex	Age	Tumor site	Imaging findings	Symptoms	IHC/Mol	Therapy	Clinical outcome	References
Negru ME et al.	2015	Italy	Mela	33 years	Sphenoid sinus	The sinonasal tract, eroding the ethmoid and sphenoid sinus with intracranial extension	Anosmia, epistaxis, reduction of visual acuity in the left eye, and headache	IHC: CD99 +, NSE+, CD56 +, Syn +, pan CK +, Vimentin +, low–molecular-weight cytokeratins +; FISH: t(11;22)	First–line chemotherapy (Doxorubicin + Isofosfamide + Mesna + Vincristine) for three cycles, followed by radiotherapy (54 Gy) and second–line chemotherapy (Cyclophosphamide + Etoposide + Mesna) for six cycles	More than 15 months, Alive	(11)
Turki S et al.	2016	Tunisia	Female	4 years	Sphenoid sinus	The sphenoid sinus with orbital and intracranial extension	Left proptosis and headache	IHC: CD99 +, CK +	Chemotherapy VIDE (Vincristine + Isofosfamide + Doxorubicin + Etoposide) for three cycles	2 months: Died	(12)
Wu KP et al.	2021	China	Mela	26 years	Sphenoid sinus	The right sphenoid sinus, extending into the right orbital apex and posterior ethmoid sinus	Intermittent right blepharoptosis and facial swelling	IHC: CD99 +, NKX2.2 +, Vimentin +, Syn +, EMA +, CK -, LCA -, CgA -, CD56 -, P40 -, CK7 -, CK5/6 -, IMP3 -, S100 -, GFAP -, HMB45, SOX10 -	Surgery, followed by chemotherapy (Vincristine, Doxorubicin and Cyclophosphamisde, alternating with Ifosfamide and Etoposide) for six cycles, and radiotherapy (5,600 cGy) and Anrotinib	More than 12 months, Alive	–
Varan A et al.	1998	Turkey	Mela	10 years	Sphenoid bone	The left greater wing of the sphenoid and destroying the bony structures of the medial wall of the zygoma and the lateral wall of the left orbit	The somnolence, left abducent nerve paralysis, and a firm, 3-cm mass antero-inferior to the left auricle	NS	Surgery	NS	(13)
Sharma RR et al.	2000	Sultanate of Oman	Female	16 years	Sphenoid bone	The greater wing of sphenoid bone on the left with extension into the left orbital cavity, middle cranial fossa, temporal fossa, and the infratemporal fossa, as well as in the retromaxillary and parapharyngeal regions	The progressive swelling in the left temporal region, proptosis, and diminished vision in the left eye	IHC: PAS +, vimentin -, desmin -, NSE -, myoglobin -, S-100 -, LCA -, GFAP -	Surgery, followed by radiotherapy (45 Gy/20 F) and chemotherapy (Cyclophosphomide + Actinomycin-D + Vincristine + Driamycin) for six cycles	More than 12 months, Alive	(14)
Singh P et al.	2002	India	Mela	4 years	Sphenoid bone	The left middle cranial fossa with destruction of the greater wing of sphenoid and temporal bone, and extension into the infratemporal fossa	The right-sided focal seizures with secondary generalization	NS	Surgery, followed by radiotherapy (35 Gy/15 F) and chemotherapy (Vincristine + Adriamycin + Cyclophosphamide) for six cycles	NS	(15)
Apostolopoulos K et al.	2003	Greece	Mela	22 years	Sphenoid bone	The greater wing of the sphenoid bone, extending to the inner wall of the left orbit, the middle cranial fossa, both sides of the sphenoid, the ethmoids, the infratemporal, and parapharyngeal space	Diplopia at regular intervals, intermittent headaches, obstruction of nasal breathing, anosmia, and reduced vision in the left eye	IHC: CD99 +.CD57 +	Chemotherapy and radiotherapy	More than 18 months, Alive	(16)
Sharma A et al.	2005	India	Mela	5 years	Sphenoid bone	The sphenoid bone, which had extradural extension into middle cranial fossa and retro-orbital extension into the left orbit	The proptosis and diminution of vision in the left eye	IHC: CD99 +, PAS +, desmin -, Syn -, chromogranin -, cytokeratin -, panactin -	Surgery, followed by radiotherapy and chemotherapy	NS	(17)
Singh GR et al.	2017	India	Mela	1 year	Sphenoid bone	The sphenoid bone with intra-cranial extension	The rapidly increasing painful swelling	IHC: CD99 +, FLI1 +, CD 56 +, CD45 -, NSE -, Syn -, S-100 -, PAS -	Surgery, followed by radiotherapy and chemotherapy (Vincristine, Doxorubicin, and Cyclophosphamisde, alternating with Ifosfamide and Etoposide) for 12 cycles	11 months, Died of sepsis	(18)

NS, not stated; FISH, Fluorescence *in situ* hybridization; IHC, Immunohistochemistry.

The limitations of this study were as follows: (i) a biopsy should be obtained for histopathological diagnosis to confirm Ewing’s sarcoma after imaging examination; (ii) the use of preoperative chemotherapy may increase the rate of complete surgical resection; and (iii) these genetic changes (EWS and FLI1) can be detected with cytogenetics, fluorescence *in situ* hybridization, polymerase chain reaction, and gene sequencing.

In conclusion, primary Ewing’s sarcoma rarely occurs in sphenoid bone and sinus. Multidisciplinary therapy consisting of an initial biopsy, complete surgical resection, aggressive combination chemotherapy, and localized radiotherapy should be acknowledged as the most beneficial treatment strategy for Ewing’s sarcoma of sphenoid bone and sinus and results in long-term survival.

## Ethics statement

Written consent for publication of this case report and the accompanying images was obtained from the patient.

## Author contributions

KW and ZL designed and wrote the report; KW, JW, YZL, and YunL collected the clinical data of radiotherapy and chemotherapy; XZ collected the clinical surgery data; DW and HW performed pathological diagnosis and collected pathological pictures; and YanL provided the imaging report and collected imaging pictures. All authors contributed equally to the article and approved the submitted version.

## Conflict of interest

The authors declare that the research was conducted in the absence of any commercial or financial relationships that could be construed as a potential conflict of interest.

## Publisher’s note

All claims expressed in this article are solely those of the authors and do not necessarily represent those of their affiliated organizations, or those of the publisher, the editors and the reviewers. Any product that may be evaluated in this article, or claim that may be made by its manufacturer, is not guaranteed or endorsed by the publisher.

## Abbreviations

CT, Computed tomography; MRI, Magnetic resonance imaging; FISH, Fluorescence *in situ* hybridization; IHC, Immunohistochemistry; RECIST, Response evaluation criteria in solid tumors; NCCN, National Comprehensive Cancer Network; CTV, Clinical target volume;GTV, Gross target volume; cGy, Centigray; CR, Complete response; PR, Partial response.
